# Biofabrication of ZnO nanoparticles using *Acacia arabica* leaf extract and their antibiofilm and antioxidant potential against foodborne pathogens

**DOI:** 10.1371/journal.pone.0259190

**Published:** 2022-01-05

**Authors:** Sumreen Hayat, Asma Ashraf, Muhammad Zubair, Bilal Aslam, Muhammad Hussnain Siddique, Mohsin Khurshid, Muhammad Saqalein, Arif Muhammad Khan, Ahmad Almatroudi, Zilursh Naeem, Saima Muzammil

**Affiliations:** 1 Department of Microbiology, Government College University, Faisalabad, Pakistan; 2 Department of Zoology, Government College University, Faisalabad, Pakistan; 3 Department of Bioinformatics and Biotechnology, Government College University, Faisalabad, Pakistan; 4 Department of Biotechnology, University of Sargodha, Sargodha, Pakistan; 5 Department of Medical Laboratories, College of Applied Medical Sciences, Qassim University, Buraydah, Saudi Arabia; Institute for Biological Research, University of Belgrade, SERBIA

## Abstract

Emergence of multidrug resistant pathogens is increasing globally at an alarming rate with a need to discover novel and effective methods to cope infections due to these pathogens. Green nanoparticles have gained attention to be used as efficient therapeutic agents because of their safety and reliability. In the present study, we prepared zinc oxide nanoparticles (ZnO NPs) from aqueous leaf extract of *Acacia arabica*. The nanoparticles produced were characterized through UV-Visible spectroscopy, scanning electron microscopy, and X-ray diffraction. *In vitro* antibacterial susceptibility testing against foodborne pathogens was done by agar well diffusion, growth kinetics and broth microdilution assays. Effect of ZnO NPs on biofilm formation (both qualitatively and quantitatively) and exopolysaccharide (EPS) production was also determined. Antioxidant potential of green synthesized nanoparticles was detected by DPPH radical scavenging assay. The cytotoxicity studies of nanoparticles were also performed against HeLa cell lines. The results revealed that diameter of zones of inhibition against foodborne pathogens was found to be 16–30 nm, whereas the values of MIC and MBC ranged between 31.25–62.5 μg/ml. Growth kinetics revealed nanoparticles bactericidal potential after 3 hours incubation at 2 × MIC for *E*. *coli* while for *S*. *aureus* and *S*. *enterica* reached after 2 hours of incubation at 2 × MIC, 4 × MIC, and 8 × MIC. 32.5–71.0% inhibition was observed for biofilm formation. Almost 50.6–65.1% (wet weight) and 44.6–57.8% (dry weight) of EPS production was decreased after treatment with sub-inhibitory concentrations of nanoparticles. Radical scavenging potential of nanoparticles increased in a dose dependent manner and value ranged from 19.25 to 73.15%. Whereas cytotoxicity studies revealed non-toxic nature of nanoparticles at the concentrations tested. The present study suggests that green synthesized ZnO NPs can substitute chemical drugs against antibiotic resistant foodborne pathogens.

## Introduction

Foodborne diseases have emerged as the major public health concerns across the globe. As reported by World Health Organization (WHO), almost 30% population is affected by foodborne illnesses in industrialized countries annually [1]. Utilization of foods contaminated with some of the foodborne pathogens such as fungi, bacteria, and viruses, is considered as a major source of foodborne diseases in human beings. *Salmonella* spp., *Escherichia coli*, *Klebsiella pneumoniae*, *Pseudomonas aeruginosa*, *Staphylococcus aureus*, *Shigella* spp., *and Listeria monocytogenes*, are the most frequently reported foodborne pathogens from different parts of the world [2]. Since 2000, an increase in drug resistance among foodborne pathogens has been reported and *Salmonella enterica* and *Escherichia coli* are among the most important pathogens exhibiting antibiotic resistance since they can do zoonotic transfer of resistant genes easily [3]. One of the primary causes of antibiotic resistance in bacteria is massive misuse of antibiotics, despite warnings regarding overuse the antibiotics are overprescribed worldwide [4]. Biofilm formation and exopolysaccharide (EPS) production also contribute towards antimicrobial resistance among pathogens [5]. Thus, development of novel and natural antibacterial agents is needed to combat emergence of multidrug resistance among commonly occurring foodborne pathogens.

Nowadays nanotechnology has established an advanced solution to overcome the problems of antimicrobial resistance by using nanoparticles (NPs). Over the past few decades, metals and its oxides occupied much interest due to their capacity of surviving insensitive conditions of different procedures [6]. Oxides of metals like zinc oxide are of special interest because of its stable, safe and non-toxic nature [7]. Nanoparticles of zinc oxide have become more attractive due to their applications in biology for example biological labeling, nanomedicine, delivery of gene, delivery of drug and biological sensing. There is also the revelation of their antifungal, antibacterial, antidiabetic and acaricidal activities [8]. ZnO NPs have wide range of antibacterial activities depending on concentration, size, and stability of NPs in the growth medium [9]. ZnO NPs mode of action is still under studies but it is assumed that their antibacterial activities are contributed to the production of hydrogen peroxide or zinc oxide nanoparticles binding to bacterial surfaces due to electrostatic force [10].

In general, various physicochemical approaches exist for metallic NPs synthesis, but certain concerns are associated with them such as cost, toxicity, complexity hinder, and environmental hazards [11]. Green synthesis of nanoparticles has been proved as one of the most eco-friendly, reliable, and cost effective approaches relying on the usage of microorganisms (bacteria, fungi) and plant extracts [12]. Plant mediated synthesis of NPs has become a center of attention for scientists and researchers globally because of its safety, wide distribution and easy availability of plants [13]. Based on the literature, ZnO NPs synthesis has been carried out by extracts of plant such as *Aloe vera* [14] *Ocimum basilicum* [15] and *Citrus aurantifolia* [16] and orange fruit juice [17]. *Acacia arabica* has been recognized globally as a multipurpose tree and commonly known as babul, kikar, or Indian gum. It has been broadly distributed throughout the arid and semi-arid zones of the world. *A*. *arabica* has been evidenced effective against malaria, sore throat, and toothache [18]. Different species of *Acacia* have been reported for the biofabrication of NPs such as *A*. *senegal* and *A*. *nilotica* [19].

In the present study we prepared ZnO NPs using aqueous leaf extracts of *Acacia arabica* and further investigated the antibacterial and antibiofilm potential of these NPs against multidrug resistant foodborne pathogens.

## Materials and methods

### Green synthesis of ZnO NPs

Fresh leaves of *Acacia arabica* were collected from the local market of Faisalabad, Pakistan. Botanical identity was authenticated by Professor Dr. Naeem Iqbal from Department of Botany, GCUF using standard keys and descriptions [20] and confirmed with the help of herbarium voucher no. GCU-302. Plant leaves were cleaned up with tap water and then distilled water to get rid of any debris. After that the leaves were air dried by keeping them at room temperature and dried leaves were ground with pestle and mortar to obtain fine powder. The aqueous extract of the plant was prepared by cold maceration technique. For this purpose, 50 grams of powdered plant was soaked in 100 ml of distilled water and the suspension was placed in a shaker at 20°C for 24 hours with continuous agitation at 100 rpm. The extract was filtered and stored at 4°C for further analysis [21].

For green synthesis of ZnO NPs, method described by Elumalai et al. [22] was used with minor modifications. The aqueous extract of plant (25 ml) was heated at 65–80°C on a magnetic stirrer. Zinc nitrate hexahydrate (Zn (NO_3_)_2_.6H_2_O) (2.5 grams) was added into the plant extract when its temperature was 60°C. The mixture was left for an hour till white precipitates appeared and was placed in hot air oven (at 60°C) for overnight until a creamy paste was formed. This paste was washed thrice with distilled water: ethanol (3:1) solution. Washed paste was added into a ceramic cup, heated at 400°C in a furnace to obtain a white powder that was stored in a sterile container for characterization and further analysis.

### Characterization of ZnO NPs

Fourier transform infrared spectroscopy (FTIR), Transmission electron microscopy(TEM), and X-ay diffraction (XRD) were used to characterize the ZnO NPs as defined by Mahamuni et al. [23]. FTIR was performed via FTIR spectrophotometer (Bruker, Massachusetts, USA) by the use of attenuated total reflectance (ATR) mode. The resulted spectrum of FTIR ranged from 4000 to 400 cm^-1^ wave number. Nanopowder of zinc oxide was suspended in ethanol, sonicated and then coated onto copper grid, dried and examined by TEM (Jeol LTD, Tokyo, Japan). The pattern of X-ray diffraction for ZnO NPs was observed by the use of X-ray diffractometer (D8 Advance diffractometer, Bruker, USA) by Cu Kα radiation of wavelength λ = 0.1541 nm in the range 2θ = 20–90° [24]. Additionally, zeta potential of biosynthesized ZnO NPs was determined using Malvern Zetasizer Nano-ZS zen 3600 (UK).

### Bacterial culture preparation

Multidrug resistant foodborne bacterial isolates (*Pseudomonas aeruginosa*, *Staphylococcus aureus* and *Salmonella typhimurium*) were collected from the Department of Microbiology, Government College University Faisalabad (GCUF), Pakistan. These multidrug resistant foodborne bacterial isolates were cultured in Muller Hinton broth (MHB) for 24 hours at 37°C with continuous agitation at 100 rpm.

### *In vitro* susceptibility testing

#### Agar well diffusion assay

Antibacterial potential of green synthesized ZnO NPs was determined by agar well diffusion assay, against foodborne pathogens such as *S*. *typhimurium*, *P*. *aeruginosa* and *S*. *aureus* [25]. Sterile deionized water was used to prepare the colloidal solution of ZnO NPs which was sonicated at 30°C for 15 minutes. Bacterial inoculum was prepared to obtain a density of 5 × 10^5^ CFU/ml by diluting each isolate and then swabbed on Muller Hinton (MH) agar medium. Wells with the diameter of 5 mm were made on agar plates and 0.1 ml of different ZnO NPs concentrations (500 μg/ml, 250 μg/ml, 100 μg/ml and 50 μg/ml) was dispensed separately into the respective wells followed by incubation at 37°C for 24 hours. The diameter of the inhibitory zone was measured in mm after incubation. The wells containing only sterile deionized water were used as negative control.

#### Evaluation of bacteriostatic and bactericidal potential of ZnO NPs

Minimum bactericidal concentration (MBC) and Minimum inhibitory concentration (MIC) of ZnO NPs were calculated following broth microdilution method of Loo et al. [26] with minor alterations. Two-fold serial dilutions of ZnO NPs (0–1000 μg/ml) were made in Muller Hinton broth in 96-wells microtitration plate. Overnight bacterial suspension (turbidity adjusted to 5 × 10^5^ CFU/ml) was dispensed in the wells of microtitration plate having varying concentrations of NPs. The plate was incubated at 37°C for 24 hours. After incubation, a redox dye nitro-blue tetrazolium chloride (NBT, 5 mg/ml) was added to evaluate the viability of bacterial cells by observing a change in the color of dye from yellow to blue. MIC was considered as the lowest concentration of NPs that was unable to change the color of dye. The experiment was performed in triplicates along with the involvement of positive (medium and bacterial culture) and negative controls (only medium).

Ten microliter of the suspension was taken from the wells showing no visible growth (MIC and higher concentrations) and plated on MH agar plates, to determine the bactericidal potential of ZnO NPs (MBC). The plates were incubated for 24 hours at 37°C. MBC was considered as the lowest concentration that completely kills the bacteria.

#### Time kill kinetic assay

Time kill kinetic assay was performed in MHB following the methods proposed by Kalishwaralal et al. [27]. ZnO NPs were dispensed into 1 ml of MHB medium containing bacterial suspension (with turbidity adjusted to 5 × 10^5^ CFU/ml) to get the final concentrations of 0 × MIC, 0.5 × MIC, 1 × MIC, 2 × MIC, 4 × MIC and 8 × MIC relative to each isolate. The cultures were incubated for the time intervals of 0,1,2,3 and 4 hours at 37°C with agitation at 100 rpm. From the tubes, 100 μl of the culture was spread on MH agar plates followed by incubation for 24 hours at 37°C. The number of bacterial colonies was quantified as CFU/ml. Each experiment was performed in triplicate.

### Antibiofilm potential of ZnO NPs

Qualitatively antibiofilm activity of ZnO NPs was determined by tube method followed by Kulshrestha et al. [28]. Overnight bacterial cultures with turbidity adjusted to 5 × 10^5^ CFU/ml were added into the tubes having 2 ml of MHB and sub-inhibitory concentrations of NPs respective to each isolate (0.25 and 0.5 × MIC) followed by incubation for 24 hours at 37°C. Negative control tube was lacking in bacterial culture whereas NPs were absent in positive control tubes. After incubation, the culture was discarded from the tubes and the attached cells were washed thrice with physiological saline (0.85% NaCl). Crystal violet dye (0.1%) was used to stain the tubes from inside and the excess dye was removed by washing the tubes gently with deionized water. The tubes were dried to observe the thin blue layer of biofilm on the tube walls.

Microtitration plate assay was used for the quantitative evaluation of biofilm formation. Briefly, 180 μl of MHB was dispensed into 96-wells microtitration plate along with 10 μl of various concentrations of NPs (0, 0.25, and 0.5 × MIC). 10 μl of overnight bacterial cultures (with turbidity adjusted to 5 × 10^5^ CFU/ml) was also added into the wells followed by incubation at 37°C for 24 hours. After incubation, optical density (OD) was measured at 620 nm to quantify the amount of planktonic cells. The growth medium was discarded and the wells were washed thrice with 0.85% NaCl. Biofilms were fixed by applying sodium acetate and then stained for 10 minutes with crystal violet dye (0.1%). The excess dye was removed by washing the plate thrice with deionized water and then air-dried. The attached cells were eluted by adding 200 μl of 95% ethanol. The absorbance of eluted sample was measured at 570 nm by using ELISA reader to quantify the cells that were able to form biofilms. Working solution and sterile growth media were used as positive and negative control respectively [29].

The percentage inhibition of biofilm was calculated using this formula:

$$Percentage\;biofilm\;inhibition=1-\frac{OD570\;of\;cells\;treated\;with\;ZnO\;NPs}{OD570\;of\;non-treated\;control}\times 100$$

### Effect of ZnO NPs on exopolysaccharide (EPS) production

EPS of each bacterial isolate was extracted following the protocol of Repatto et al. [30] with minor modifications. Briefly, 2 ml of overnight bacterial cultures with turbidity adjusted to 5 × 10^5^ CFU/ml was dispensed into flasks having 100 ml of MHB supplemented with sub-inhibitory concentrations (0.5 × MIC) of NPs respective to each isolate. The flasks having bacterial inoculum only served as positive control. The flasks were placed at 37°C on shaking incubator for 24 hours. Following incubation, bacterial cultures were harvested by centrifugation at 6000 rpm at 4°C for 30 minutes. The pellet was discarded and the supernatant was placed into separate cryogenic vials. The EPS was precipitated by dispensing two volumes of acetone in each vial followed by overnight refrigeration at 4°C. In order to obtain EPS, the mixture was centrifuged at 6000 rpm at 4°C for 30 minutes. Wet pellet weight was measured and after drying the pellet in hot air oven at 40°C for 24 hours, the dry weight was determined.

### Antioxidant potential of ZnO NPs

The antioxidant activity of NPs was determined by 2,2-diphenyl-1-picrylhydrazyl (DPPH) radical scavenging assay following the method of Pallavicini et al. [31]. Different concentrations of ascorbic acid and NPs (25 to 100 μg/ml) were prepared and 3 ml of each concentration was added into 2.96 ml of 0.1 mM DPPH solution i.e., prepared in methanol. These mixtures were continuously stirred and kept in dark for 20 minutes. A color change from purple/ violet to yellow indicated the presence of antioxidant activity. The absorbance of reaction mixture was read at 517 nm using spectrophotometer. 0.1 mM DPPH solution was used as control while ascorbic acid was used as a standard solution. Percentage DPPH radical scavenging activity was determined by following formula.

DPPH scavenging activity (%) = [(Abs_control_−Abs_sample_) / (Abs_control_)] × 100

Abs_control_ (absorbance of DPPH + ascorbic acid)

Abs (absorbance of DPPH + sample/nanoparticles)

### Cytotoxic potential of ZnO NPs

Neutral red uptake assay was used to access the cytotoxic potential of ZnO NPs following the method of Selim et al. [32] using HeLa cell lines. Dulbecco’s modified Eagle’s high glucose medium having 10% FBS and 1% antibiotics (penicillin/streptomycin) was used to grow cell lines. The media was incubated for 24 hours at 37°C with 90% humidity and 5% CO_2_. After that, the media was changed and incubated with various NPs concentrations ((0–100 μg/ml) again for 24 hours. The growth medium lacking NPs served as a control. The growth medium was discarded after incubation and the cells were washed with PBS (Phosphate buffer saline) twice. In each well, neutral red media (100 μl) was added and plates were incubated again for 2 hours at 37°C. After incubation, neutral red media was removed and the cells were washed with PBS again and detained with 150 μl of fresh detaining solution (50% ab. ethanol, 49% water and 1% glacial acetic acid). To shake the plate microtiter plate shaker was used for 10 minutes followed by measurement of absorbance at 540 nm. The Charge-Coupled System (CCD) SP 480 H with colour camera (Olympus, Japan) was used to perform phase contrast microscopy of HeLa cells (treated and controlled).

### Statistical analysis

All the experiments were performed in triplicates and the obtained data were tabulated in Microsoft™ excel spread sheets and analysed using IBM™ SPSS Statistics 25.0 (IBM™ Corporation, Armonk, NY, USA). Means and standard deviations were calculated for the numerical data like EPS quantification and % age scavenging activity of ZnO nanoparticles against food borne pathogens. Frequencies and percentages were calculated for categorical variables like time kill kinetics of pathogens with different ZnO nanoparticles concentrations. One way ANOVA (Analysis of Variance) and Student’s t-test was used for the comparison of means and p≤0.05 value was considered as significant.

## Results and discussion

Resistance exhibited by different foodborne pathogens against commonly used antibiotics has become a primary concern to public health across the globe. Moreover, biofilm formation is also linked to an increase in bacterial resistance to conventional antibiotics. Metal oxide nanoparticles have been proved as an effective strategy against antibiotic-resistant bacteria by overcoming the problems associated with the emergence of resistance. Synthesis of nanoparticles can be carried out by various physicochemical methods, but currently green synthesis approach has been appreciated because of its safety, eco-friendliness, cost-effectiveness, and reliability [12]. In the present study, we also synthesized ZnO NPs via green technology by using aqueous leaf extracts of *A*. *arabica*. Plant extracts have been considered as promising sources to produce NPs since they are rich in antimicrobial and antioxidant compounds that can also be used as reducing and capping agents during the green synthesis of NPs [31].

### Characterization of ZnO NPs

**Fig 1:**
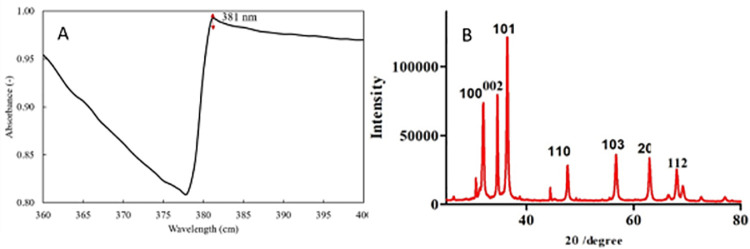
Absorption spectra using UV-Vis spectrophotometer (A) XRD pattern (B) of green synthesized ZnO NPs.

In this study, rod-shaped ZnO NPs were synthesized with average size of 20 nm. UV-vis spectroscopy of ZnO nanoparticles was performed to monitor the synthesis of NPs. The absorption peak was recorded at 381nm, corresponding to pure ZnO and no other peak was found in the spectrum revealing the presence of only ZnO (Fig 1A). The X-ray diffraction (XRD) pattern of green synthesized ZnO NPs has been shown in Fig 1B. The XRD spectra revealed well defined diffraction peaks having 2θ values at 31.72°, 34.39°, 36.23 and 47.44° with reflection planes (100, 002, 101, and 102) (Fig 1B) corresponding well with the hexagonal/rod structure of ZnO nanoparticles prepared by green synthesis method. These peaks are in accordance with Yildiz et al. [33] who also reported the rods/hexagonal structure of ZnO nanoparticles.

**Fig 2:**
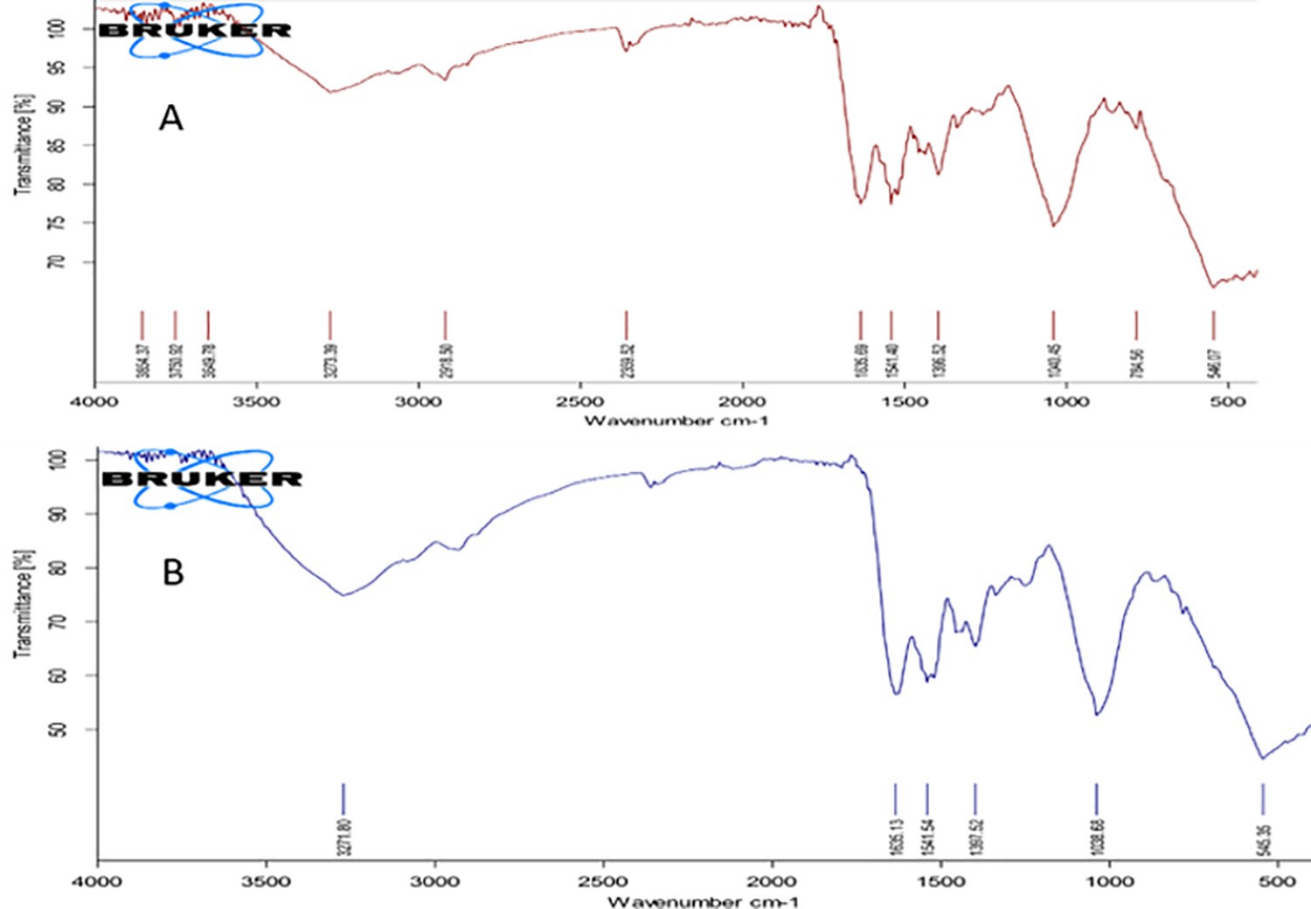
FTIR spectra of *Acacia arabica* extract (A) green synthesized ZnO NPs by *Acacia arabica* extract (B).

In order to assess various phytochemicals responsible for the synthesis and stabilization of nanoparticles, the Fourier transform infrared spectroscopy (FTIR) spectrum of green synthesized ZnO was conducted. The results showed that the peaks around 545.35 cm^−1^ were corresponding to ZnO [34]. While, the peak at 3443 cm^−1^ might be related to the free hydroxyl group of phenols responsible for the capping of nanoparticles [35], and the peaks at 2922 cm^−1^ revealed the presence of–CH stretching [36]. The peaks around 1586 cm^−1^ and 1412 cm^−1^ were due to the amide groups. Due to atomic vibration, metal oxides generally exhibit an absorption peak below 1000 cm^−1^ [37]. Free carboxylate groups are supposed to bind to nanoparticles (metallic) in order to make them stable [24]. Some minor changes in absorption peaks positioned between the FTIR spectrum of the plant extract and green synthesized ZnO NPs were observed (Fig 2A and 2B). FTIR analysis indicated many phytoconstituents in *Acacia arabica* that were responsible for ZnO nanoparticle synthesis. Plant extract compounds including OH, amino, and CO groups have been reported to play a promising role in reduction and stabilization of nanoparticles [38].

**Fig 3:**
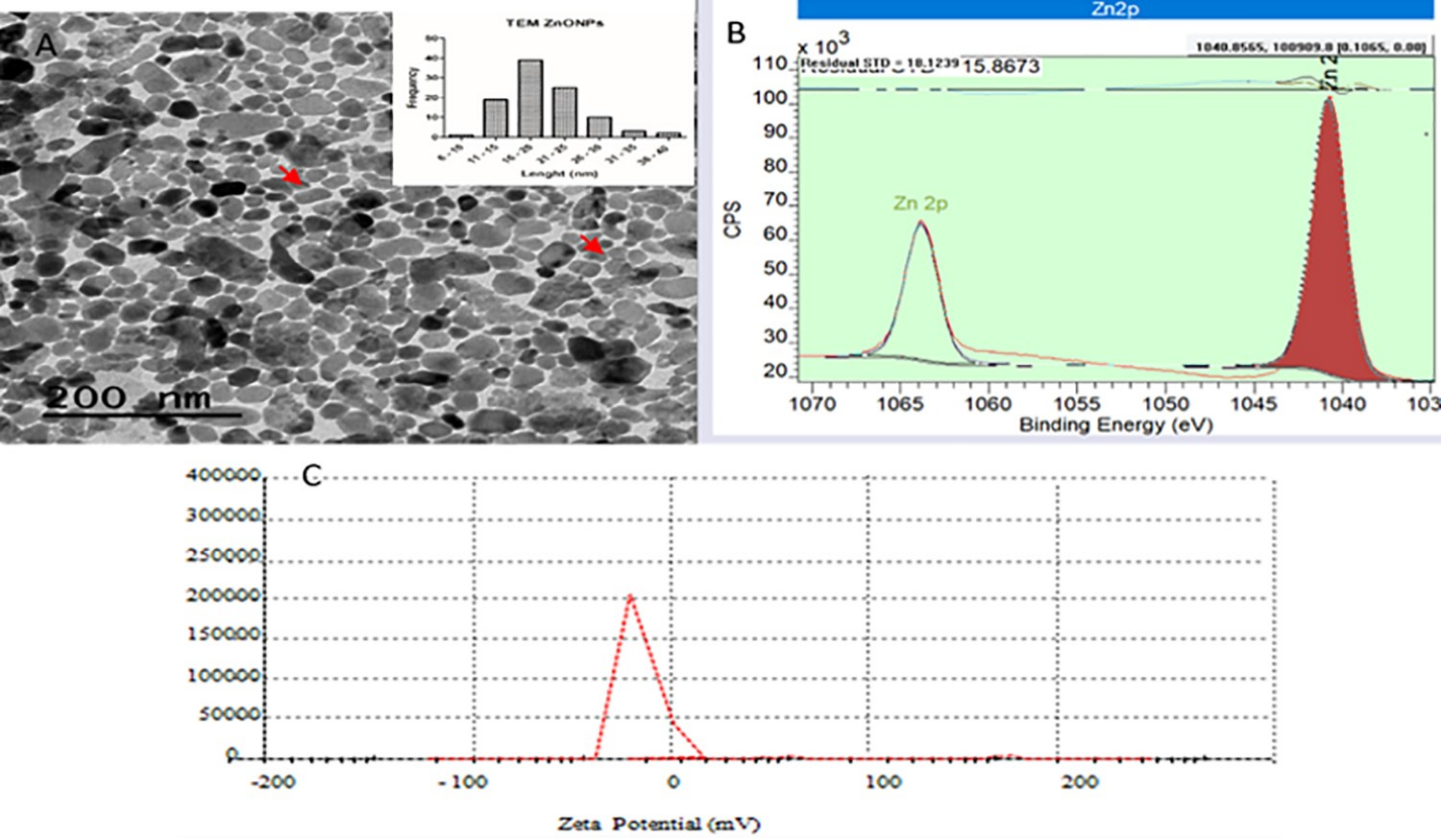
TEM image of the ZnO NPs showing rod-shaped particles without aggregation (A) The average size by XRD spectrum (B) zeta potential of ZnO NPs (C).

The shape and size of the nanoparticles was determined by TEM. Most of the ZnO NPs were rod-shaped without aggregation having diameter of 16–20 nm (Fig 3A). The average calculated size by XRD spectrum was 11.3 nm (Fig 3B). Zeta potential is a measure of effective electric charge on the particle surface and it controls the particles stability. However, it measures the electrical potential some distance away from the particle surface and is influenced with solution pH, solvent composition and ionic strength [39]. Aggregation of the particles and zeta potential are also influenced with nature of the solvent [40]. A hydrophobic solvent film leads to repulsion thereby making maximum contact with water. In our case, solvent used was milli Q water which exhibits amphipathic nature and has no effect on the charge distribution. The stability of ZnO NPs was monitored by zeta potential as shown in Fig 3C. The negative zeta potential indicates that the particle surface is negatively charged due to the presence of OH, carboxylic groups etc. which is also evident from the FTIR spectrum. The presence of negative charges on particle surfaces favors the selectivity and sequestration of cationic contaminants [41]. Similar results were obtained by Agarwal et al. [42] using tomato leaf extract and Nithya and Kalyanasundharam [43] using *Cardiospermum halicacabum* leaf extracts for the biosynthesis of ZnO particles. They reported mean zeta potential (-20.9 mV and -32-06 t0 17.89 mV) of the particles with moderate stability.

### *In vitro* susceptibility testing

It has been well reported in the literature that ZnO NPs show strong antibacterial potential against Gram-negative and Gram-positive bacteria [44]. In this study, the antibacterial activity of green synthesized ZnO NPs was assesed against different multidrug resistant foodborne pathogens such as *S*. *aureus*, *S*. *enterica*, and *E*. *coli* by agar well diffusion method. For well diffusion assay clear zone formation around the well indicated antibacterial effect. The results demonstrated that NPs exhibited a dose-dependent antibacterial activity and that the diameter of inhibitory zone increased with an increase in nanoparticles concentration. The diameter of the zone of inhibition ranged from 16–30 nm. Maximum inhibition was observed against *E*. *coli* at 500 μg/ml of nanoparticles where zone of inhibition diameter was 30 nm, while the minimum inhibitory effect was observed for *S*. *aureus* (26 nm) at the highest concentration tested (S1 Data). One of the previous studies by Naseer et al. [45], also reported that Gram-negative bacteria were more susceptible to biogenic ZnO NPs as compared to *S*. *aureus* due to the presence of a thick layer of peptidoglycan in the cell wall of Gram-positive bacteria. In another study, by Cox et al. [46], biogenic ZnO NPs from leaf extract of *Melia azadarach* exhibited an inhibitory zone of 32 nm against *S*. *aureus* at a concentration of 1000 μg/ml. There are number of factors contributing towards the antibacterial activities of ZnO NPs such as size of NPs, shape, zeta potential, concentration, specific surface area etc. It was observed that ZnO NPs with positive surface potential exhibited significantly greater antimicrobial activities when compared with NPs having same size but with negative surface potential [47]. Moreover, small sized NPs can easily penetrate bacterial membrane when compared with NPs having large diameter [48].

Disc diffusion/ well diffusion methods are documented as preliminary tests to check the antimicrobial activities of various therapeutic agents; therefore, a further evaluation of antibacterial activities of ZnO NPs by MIC determination was required [49]. Using broth microdilution assays, MIC and MBC values were measured. The results revealed that MIC values of ZnO NPs against *S*. *aureus*, *S*. *enterica*, and *E*. *coli* were 62.5, and 31.25 μg/ml respectively, whereas MBC values were found to be 62.5 μg/ml for all tested bacteria (S1 Data). Similarly, Atkinson et al. [50], reported MIC value of 50 μg/ml of biogenic ZnO NPs against *S*. *aureus* and *E*. *coli*. In contrast to our study, Vanaja et al. [51], reported MIC and MBC values of 3.9 and 7.81 μg/ml respectively against *S*. *aureus* using ZnO NPs as an antibacterial agent to treat mastitis in sheep.

### Time kill kinetic assay

**Fig 4:**
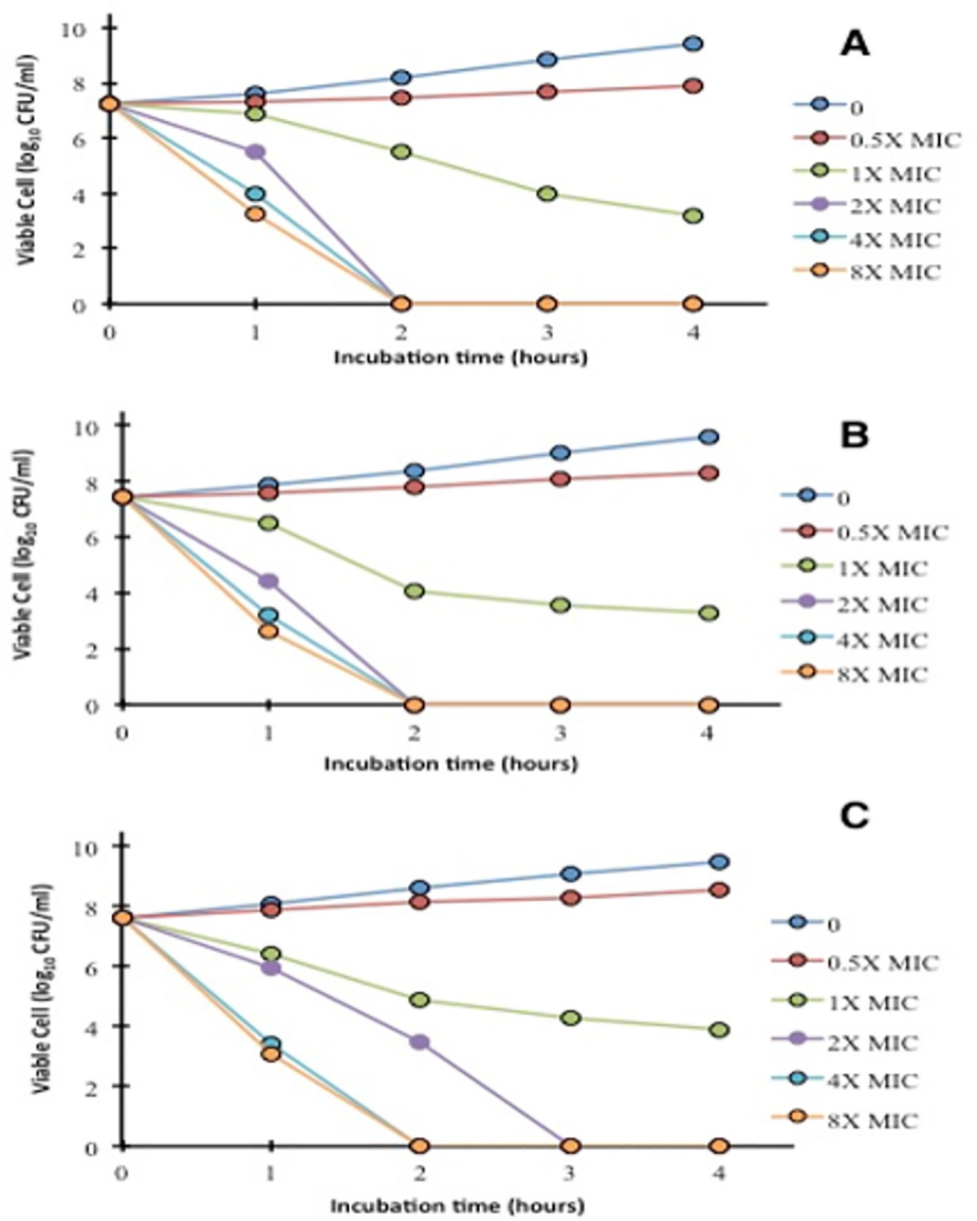
Time kill kinetics of antibiotic-resistant foodborne pathogens (A) *S*. *aureus* (B) *S*. *enterica* (C) *E*. *coli*, after treatment with different concentrations of green synthesized zinc oxide nanoparticles.

**Fig 5:**
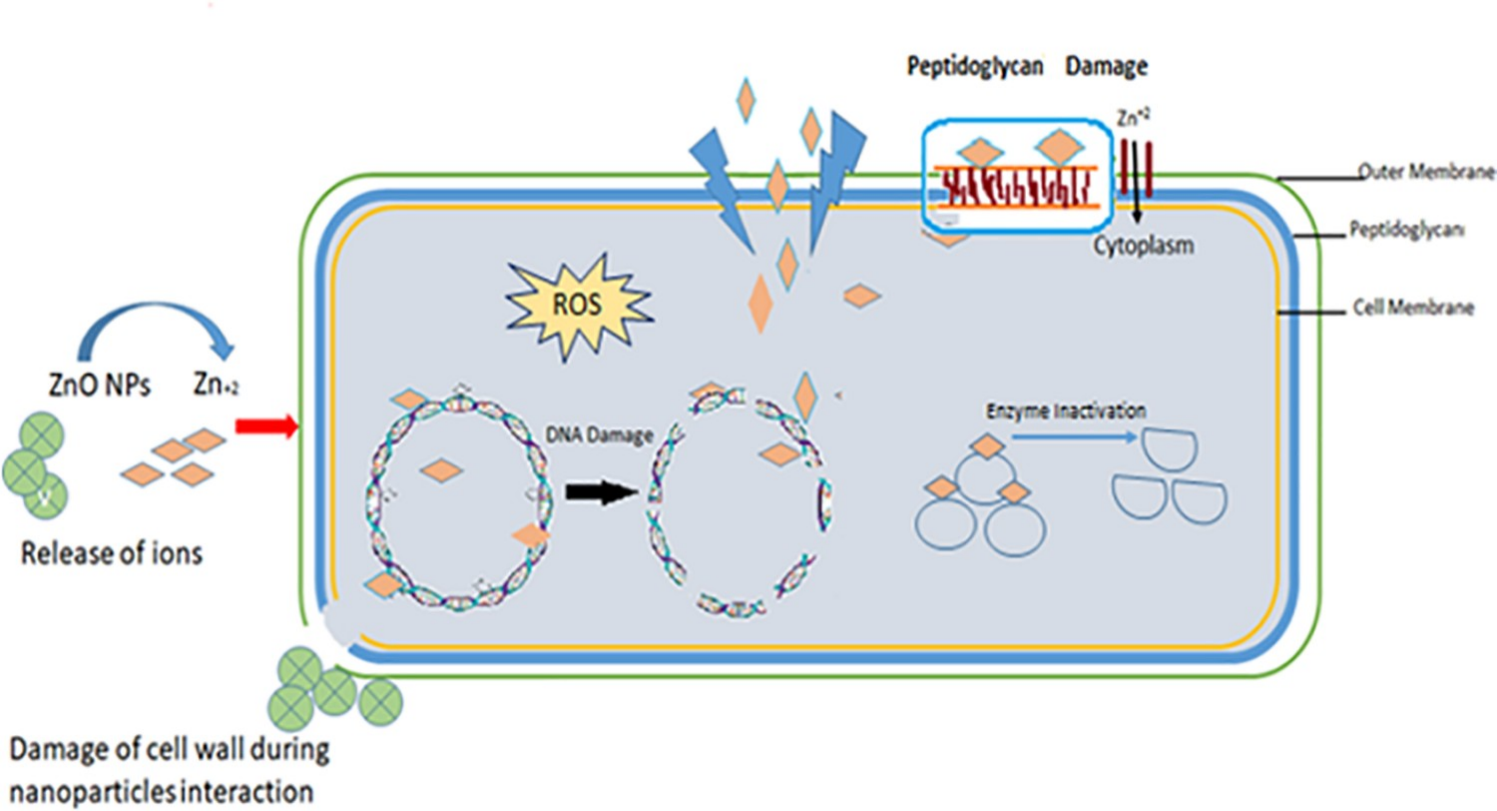
A schematic diagram of the antibacterial activity mechanism.

One of the major factors in bacterial pathogenesis is its rapid reproduction rate that can be efficiently targeted to prevent viable bacterial infections [52], as ZnO NPs did in this study and the bactericidal effect was observed in a dose-dependent manner. Time-kill kinetics of antibiotic-resistant foodborne pathogens after treatment with different concentrations (0, 0.5, 1, 2,4, 8 × MIC) of ZnO NPs has shown in Fig 4. These results confirmed the bactericidal potential of green synthesized ZnO NPs, and it was observed that the bactericidal endpoint for *S*. *aureus* and *S*. *enterica* reached after 2 hours of incubation at 2 × MIC, 4 × MIC, and 8 × MIC. While for *E*. *coli*, the bactericidal endpoint was reached after 3 hours of incubation at 2 × MIC, and after 2 hours of incubation at 4 × MIC and 8 × MIC of NPs (Fig 4A–4C). No significant differences in reduction in viable counts were observed in tested bacterial isolates (p > 0.05). Several mechanisms have been proposed for antimicrobial activities of ZnO NPs such as the generation of hydrogen peroxide or building up of ZnO particles on the cell surface of bacteria [38], reactive oxygen species (ROS) generation, release and internalization of zinc ions that will ultimately lead to the membrane disruption and cell damage [53, 54]. Fig 5 shows different mechanisms adopted by bacterial cell for ZnO NPs attachment and entrance inside the bacteria. The interaction between released zinc ions and the bacterial cell wall results in membrane damage as shown in Fig 5 at the point of contact. Production of ROS molecules such as superoxide, hydroxyl and peroxide ions on the surface of nanoparticles could enhance the antimicrobial activity.

### The antibiofilm potential of ZnO NPs

The formation of biofilms plays a vital part in the bacterial pathogenesis, meanwhile it is also involved in food contamination and spoilage. Biofilm development is monitored and controlled by a signal-mediated quorum sensing phenomenon [55]. Biofilm formers as compared to their planktonic counterparts have exhibited inherent recalcitrance to antibiotics. This is one of the reasons for difficulty in treating infections resulting by formation of biofilm in bacteria [35]. Sub-inhibitory effect of zinc oxide nanoparticles concentrations (0.25 and 0.5 × MIC) on biofilm formation was evaluated qualitatively by tube method in which bacteria were cultured in different concentrations of green synthesized ZnO NPs. The thin layer of developed biofilms was observed after staining the adhered cells with crystal violet dye. The results revealed that the formation of a thin layer of biofilms was reduced in a concentration dependent manner. As depicted in S2 Data, biofilm formation was significantly reduced in all the tested bacteria treated with ZnO NPs (at concentrations of 0.5 × MIC) in comparison with positive control cells. Our results are in accordance with the findings of Siddique et al. [55] who also reported no visible biofilm formation by *Klebsiella pneumoniae* after treatment of cells with silver nanoparticles at concentration of 100 μg/ml.

**Fig 6:**
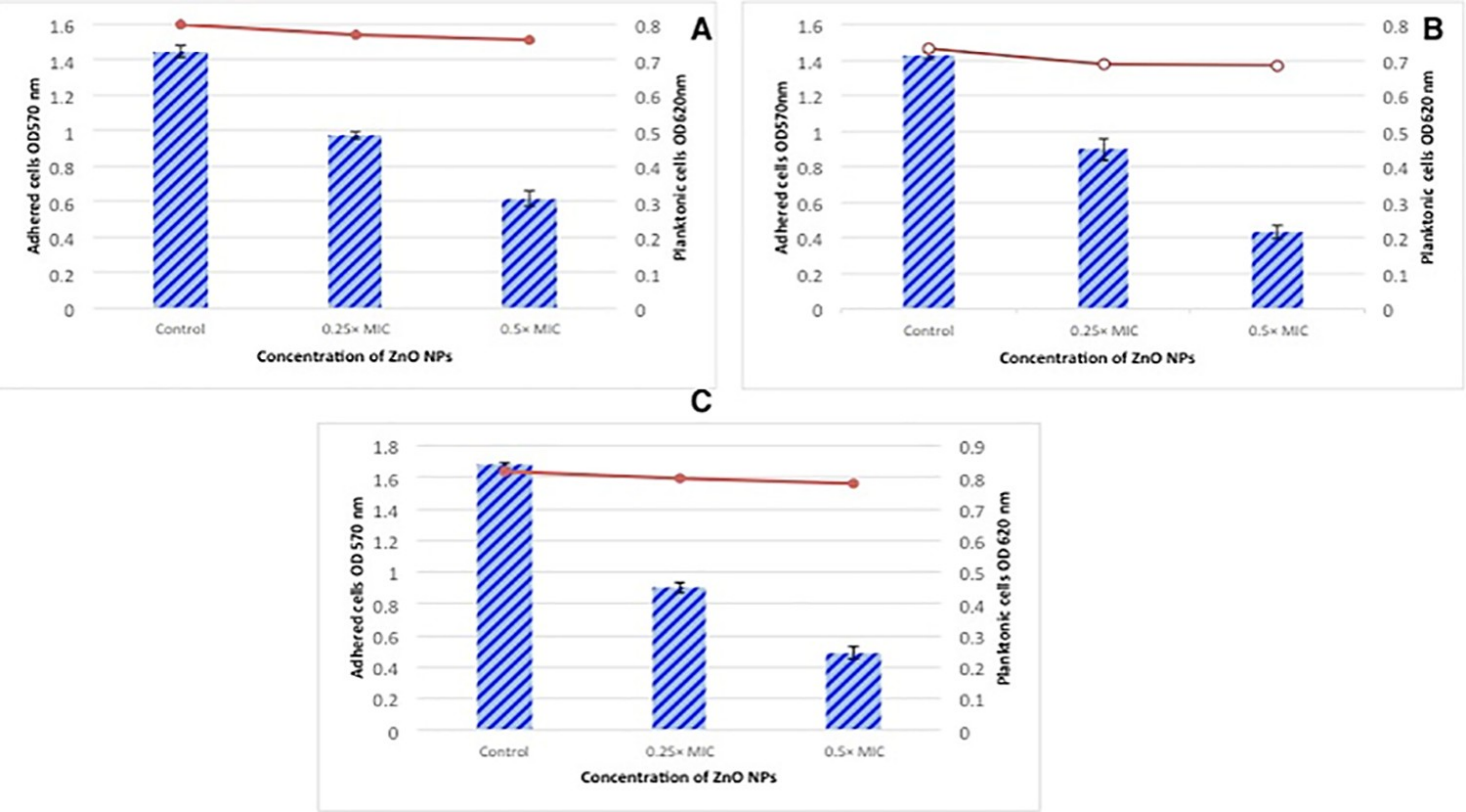
Antibiofilm activities of green synthesized ZnO NPs against multidrug-resistant foodborne pathogens (A) *S*. *aureus*, (B) *S*. *enterica*, (C) *E*. *coli*.

For the quantitative evaluation of biofilm formation, microtiter plate assay was performed, and the results showed a significant increase (p < 0.05) in percentage inhibition of biofilms formation after treatment of cells with sub-inhibitory concentrations of green synthesized ZnO NPs. After 24 hours of incubation, maximum biofilm formation in untreated cells was observed for *E*. *coli*, whereas the minimum number of adhered cells was found for *S*. *aureus*. As Illustrated in the Fig 6A, percentage inhibition of biofilm formation was 32.5–71.0% in the presence of NPs and maximum inhibition was observed for *E*. *coli* at 0.5 × MIC concentration of ZnO NPs (Fig 6C). Similar observations of inhibition of biofilm biomass in a dose-dependent manner by green synthesized ZnO NPs were reported by Al-Shabib et al. [56] against human and foodborne pathogens. Their data revealed 32–84% inhibition of biofilm in *P*. *aeruginosa* after treatment with various sub-inhibitory concentrations of ZnO NPs.

Sub-inhibitory concentrations of antimicrobial drugs are crucial since they donot kill the cells but alter various structural and physicochemical attributes such as surface hydrophobicity, motility, adhesion, host-bacterial interaction i.e. phagocytosis and release of ROS from phagocytes [57]. As shown in the Fig 6A–6C, the growth of bacterial cells was not significantly inhibited in the presence of subinhibitory concentrations of green synthesized NPs.

The antibiofilm potential was checked by culturing the cells for 24 hours in the presence of sub-inhibitory concentrations of NPs. Bars indicated biofilms formation whereas lines indicated the growth of bacterial cells.

### Effect of ZnO NPs on exopolysaccharide (EPS) production

**Fig 7:**
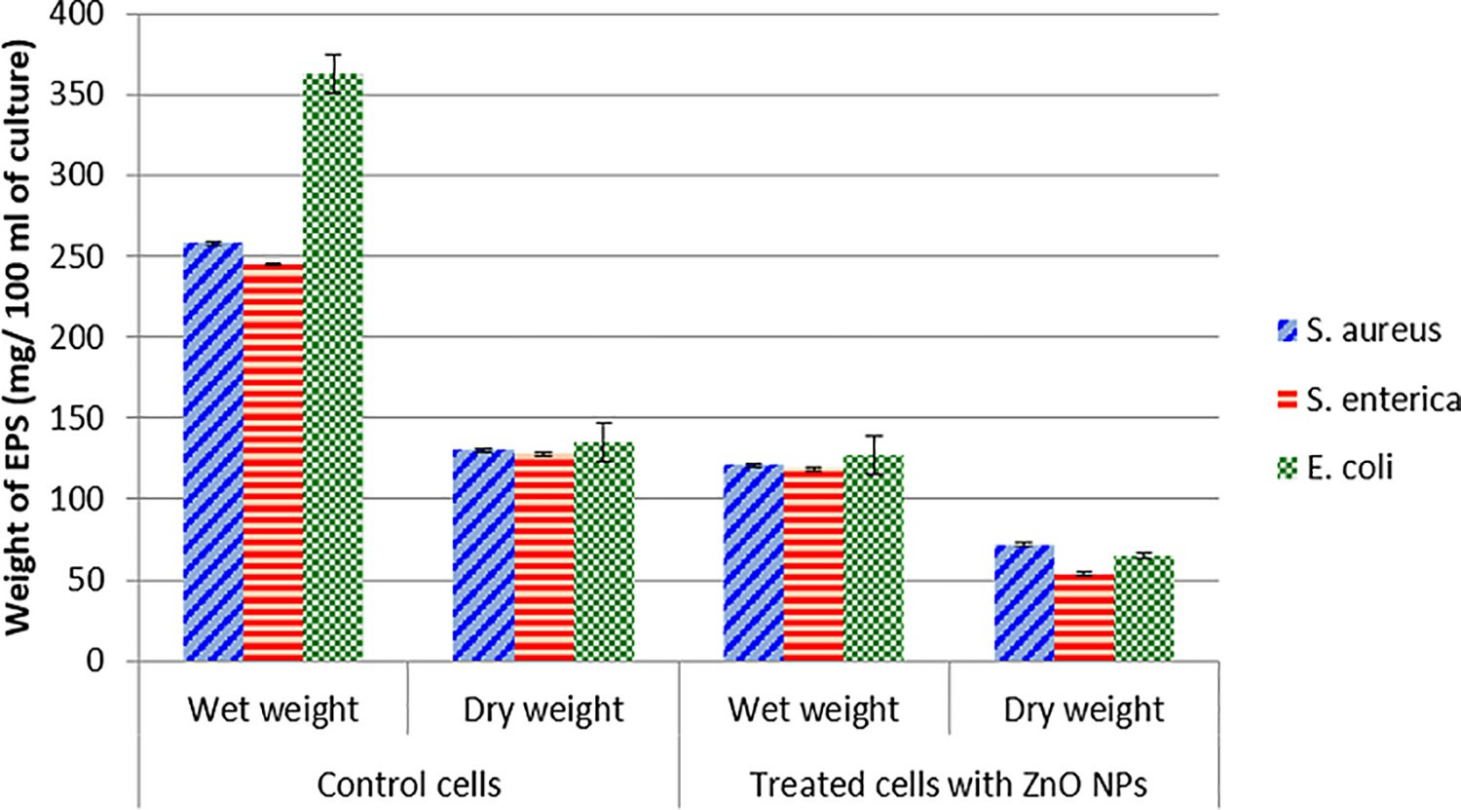
Quantification of EPS extracted from cells of antibiotic-resistant foodborne pathogens grown in the presence and absence of sub-inhibitory concentrations of green synthesized ZnO NPs. Values are presented as Mean ± Standard error; each experiment was performed in triplicates.

EPS matrix produced by microorganisms promotes the cellular organization into microbial communities (biofilms), increases the capacity of biofilm formers to trap nutrients, promotes the solubilization of hydrophobic organic compounds, coordinates cell-cell communication, facilitates gene exchange, and provides physical stability [58]. EPS also serves as a defensive barrier by preventing the entry of antibiotics into bacterial cells and increasing the resistance to antibacterial agents [46]. Enhanced production of EPS will result in an increased resistance to antibacterial agents due to the alteration of the architecture of biofilms [24]. By targeting this matrix, biofilm cells will be exposed and can easily be removed or killed by antimicrobial agents. To check the effect of green synthesized ZnO NPs on extracellular polysaccharides production of foodborne bacterial isolates, EPS was extracted, and its dry and wet weight was measured. The results showed a significant (p < 0.05) reduction in EPS production in bacterial cells treating with NPs in comparison with control cells. The data indicated that in control cells, the extracted EPS wet weight was in the range of 245–363 mg/ 100 ml of bacterial culture, whereas, dry weight of control cells was 128–135 mg/100 ml of culture. It was also observed that in the presence of NPs, wet weight was reduced to 50.6–65.1% while reduction of dry weight was 44.6–57.8% among tested bacteria (Fig 7). Muzammil et al. [29] also reported a 32.9–41.5% reduction in wet weight and 48.1–56.7% reduction in dry weight of EPS extracted from *Acinetobacter baumannii* after treatment of cells with sub-inhibitory concentrations of aluminum oxide nanoparticles. In another study by Azam et al. [37], bio-fabricated ZnO NPs also reduced the EPS production in human and foodborne pathogens and their results demonstrated that 81, 69, 68%, 67 and 59% decrease in EPS content was observed for *P*. *aeruginosa*, *E*. *coli*, *S*. *marcescens L*. *monocytogenes*, *and K*. *pneumonia*e respectively.

### Antioxidant potential of ZnO NPs

**Fig 8:**
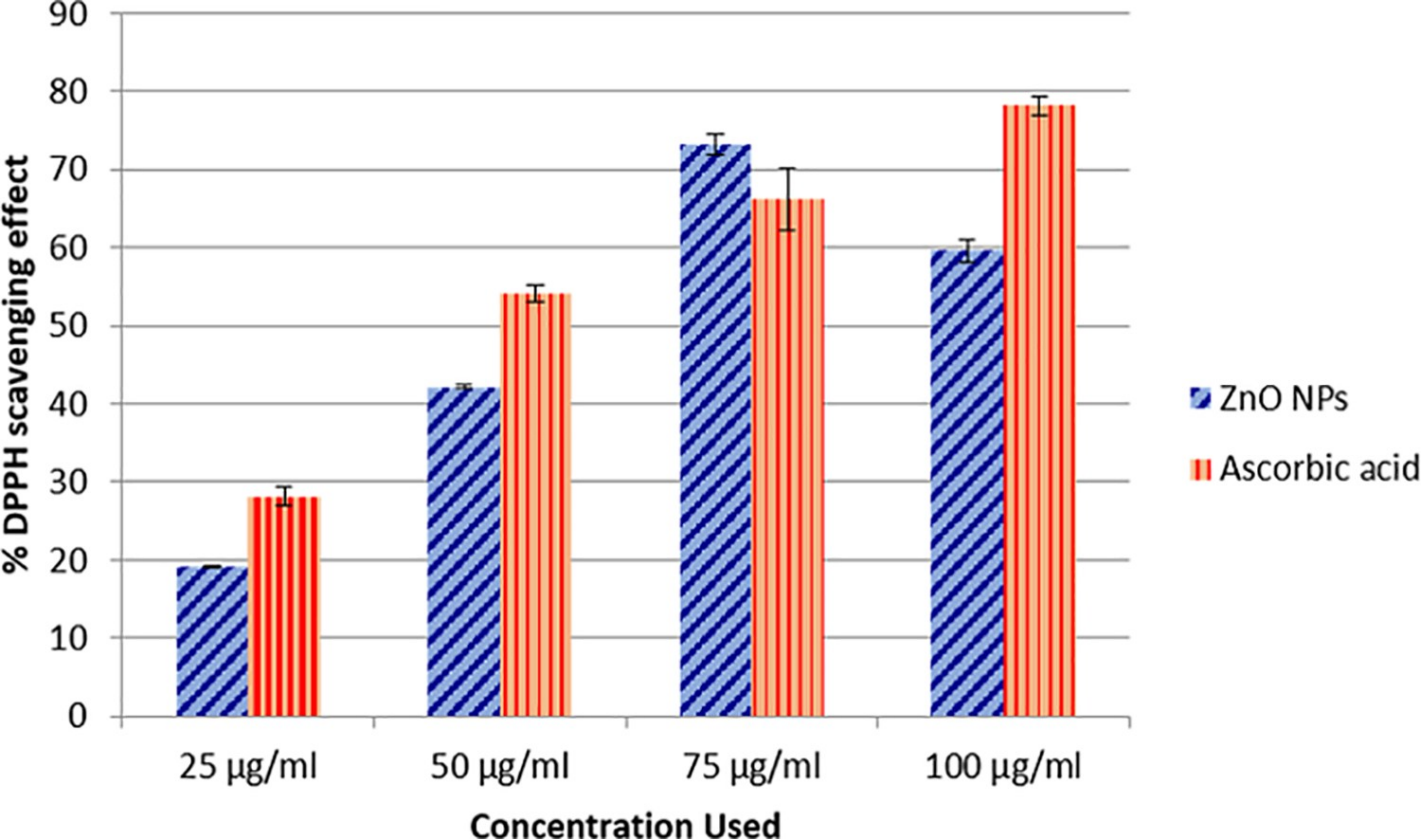
% scavenging activity of green synthesized zinc oxide nanoparticles (ascorbic acid was used as standard). Values are presented as Mean ± Standard error; each experiment was performed in triplicates.

DPPH free radical scavenging is an accepted method to screen the antioxidant activities of numerous compounds. This method has been extensively used to predict antioxidant activities since it requires a shorter time for analysis. The effect of antioxidants on DPPH is considered to be due to their hydrogen donating ability [25]. In this study, the DPPH radical scavenging assay was also used to evaluate the antioxidant potential of green synthesized NPs. It was observed that the radical scavenging potential of ZnO NPs decreased by increasing the concentration of nanoparticles. A noteworthy decrease (p < 0.05) was observed at the highest concentration used (100 μg/ml) when compared with the radical scavenging activity of ascorbic acid. The values of % scavenging potential ZnO NPs ranged from 19.25 to 73.15% as shown in the Fig 8. Our study is consistent with the findings of Loo et al. [26], who also reported an effective free radical inhibition by biogenic ZnO NPs. Their study suggested reduced radical scavenging activity of nanoparticles with increased concentrations probably because of the lower solubility of nanoparticles and insufficient DPPH content. It was observed that the maximum scavenging activity was found to be 94.55% at 25 μg/ml. In contrast to our observations, Jacob and Rajiiv [38], revealed that the antioxidant potential of ZnO NPs increased by increasing the concentration of NPs and the highest antioxidant activity was achieved at the concentrations of 200. μg/ml. Another study by Bharathi and Bhuwaneshwari [59] also showed dose dependent radical scavenging potential of ZnO NPs synthesized from rutin. The radical scavenging potential of ZnO NPs might be attributed to the presence of phenols having the capacity to donate H in their OH groups [60].

### Cytotoxic potential of ZnO NPs

**Fig 9:**
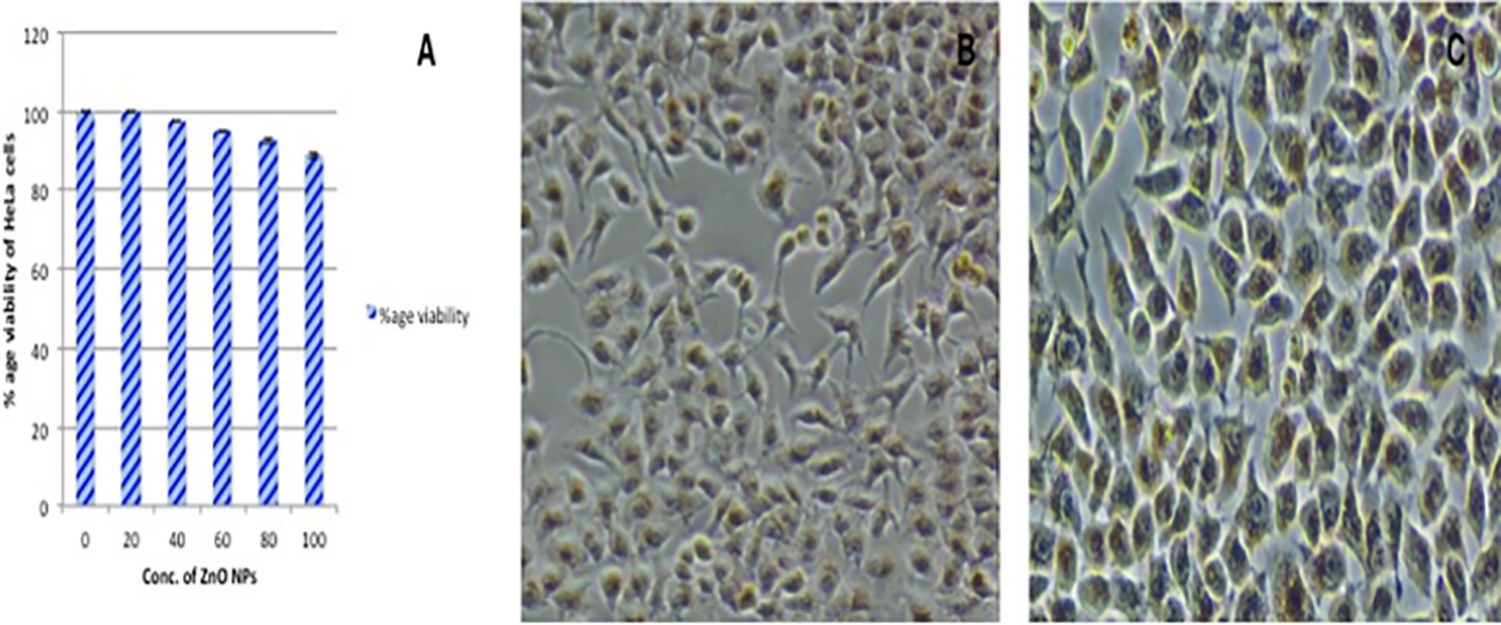
Cytotoxicity assay for ZnO NPs using HeLa cell lines (A) Percentage viability of HeLa cells treated with different concentrations of ZnO NPs (0–100 μg/ml). 100% of cell viability indicates zero toxicity. (B) Phase-contrast microscopy of untreated cells (control) (C) Cells treated with ZnO NPs at a concentration of 60 μg/ml.

Neutral red uptake assay was used to assess the cytotoxic effects of ZnO NPs on the viability of HeLa cell lines. The results showed that, relative to control cells, there was no substantial difference (p > 0.05) in the percentage viability of cells treated at different concentrations (0–100 μg / ml) of NPs (Fig 9A). The percentage viability of HeLa cells was found to be 88.6% at the highest concentration tested. Phase-contrast microscopy revealed that in untreated cells confluent monolayer was developed within 24–48 hours and > 90% of the cells displayed proper morphology (Fig 9B). The results also confirmed the non-toxic nature of ZnO NPs at the concentrations tested as no significant changes in the cell morphology were observed such as apoptosis, cell’s shrinkage and detachment (Fig 9C). It has been observed that the cytotoxicity of metallic NPs is largely dependent on the size, shape, and capping agents used during the production process [59]. The exact mechanism of cytotoxic effects has not yet been established, but it has been proposed that cytotoxic effects of ZnO NPs are mainly due to the production of dissolved zinc ions along with ROS induction. Toxicity of ZnO nanoparticles on HeLa cell lines is dose dependent as described by [61]. Many studies are demonstrating the high cytotoxic potential of ZnO NPs, whereas other reports revealed their non-toxic nature as in our study. These variations can be attributed to the differences in the number of NPs used or changes in cellular density [62]. In contrast to our observations, Bisht and Rayamajhi [61], reported a significant cytotoxic potential of green synthesized ZnO NPs in a concentration-dependent manner against HeLa cell lines.

## Conclusion

In conclusion, the findings of the present study demonstrated that biofabricated ZnO NPs using *A*. *arabica* leaf extract could be effectively used as promising antibacterial and antibiofilm agents against multidrug resistant foodborne pathogens. Moreover, the nontoxic nature of ZnO nanoparticles could be beneficial against biofouling and for the prevention of food contamination. In future, molecular studies to analyze the exact mechanism of action of ZnO NPs are required against various multidrug resistant biofilms formers.

## Supporting information

S1 Data (XLSX)

S2 Data (XLSX)

S3 Data (XLSX)

## References

[1] Organization WH. WHO initiative to estimate the global burden of foodborne diseases, fourth formal meeting of the Foodborne Disease Burden Epidemiology Reference Group (FERG) Sharing New Results, Making Future Plans, and Preparing Ground for the Countries. 2014.

[2] Siddique MH, Qamar MU, Hayat S, Aslam B, Nadeem H, Hussain S, et al. Polymicrobial multidrug-resistant bacteria isolated from street vended fresh fruit juices in Pakistan. Brit. Food J. 2018; 120: 1358–1365.

[3] Cantón R, Novais A, Valverde A, Machado E, Peixe L, Baquero F et al. Prevalence and spread of extended‐spectrum β‐lactamase‐producing Enterobacteriaceae in Europe. Clin Microbiol Inf. 2008; 4: 144–153 doi: 10.1111/j.1469-0691.2007.01850.x 18154538

[4] Rai MK, Deshmukh S, Ingle A, Gade A. Silver nanoparticles: the powerful nanoweapon against multidrug‐resistant bacteria. J App Microbiol. 2012; 112: 841–852. doi: 10.1111/j.1365-2672.2012.05253.x 22324439

[5] Abebe GM. The Role of Bacterial Biofilm in Antibiotic Resistance and Food Contamination. Int J Microbiol. 2020; 2020: 1–10. doi: 10.1155/2020/1705814 32908520 PMC7468660

[6] Ahmad A, Mukherjee P, Senapati S, Mandal D, Khan MI, Kumar R et al. Extracellular biosynthesis of silver nanoparticles using the fungus Fusarium oxysporum. Colloids Sur B. 2003; 28: 313–318.

[7] Kakati M, Das D, Das P, Sanjeev A, Kumar VS. Lett App Nanobioscience. 2014; 9: 779–783.

[8] Rehana D, Mahendiran D, Kumar RS, Rahiman AK. *In vitro* antioxidant and antidiabetic activities of zinc oxide nanoparticles synthesized using different plant extracts. Biopro Biosyst Eng. 2017; 40: 943–957. doi: 10.1007/s00449-017-1758-2 28361361

[9] Raghupathi KR, Koodali RT, Manna AC. Size-dependent bacterial growth inhibition and mechanism of antibacterial activity of zinc oxide nanoparticles. Langmuir. 2011; 27: 4020–4028. doi: 10.1021/la104825u 21401066

[10] Kathiresan K, Manivannan S, Nabeel M, Dhivya B. Studies on silver nanoparticles synthesized by a marine fungus, Penicillium fellutanum isolated from coastal mangrove sediment. Colloids Sur B. 2009; 71: 133–137. doi: 10.1016/j.colsurfb.2009.01.016 19269142

[11] Kahsay MH, RamaDevi D, Kumar YP, Mohan BS, Tadesse A, Battu G, et al. Synthesis of silver nanoparticles using aqueous extract of Dolichos lablab for reduction of 4-Nitrophenol, antimicrobial and anticancer activities. OpenNano. 2018; 3: 28–37.

[12] Zhang X, Yan S, Tyagi R, Surampalli R. Synthesis of nanoparticles by microorganisms and their application in enhancing microbiological reaction rates. Chemosphere. 2011; 82: 489–494. doi: 10.1016/j.chemosphere.2010.10.023 21055786

[13] Jadoun S, Arif R, Jangid NK, Meena RK. Green synthesis of nanoparticles using plant extracts: a review. Env Chem Lett. 2020; 1–20.

[14] Salam HA, Sivaraj R, Venckatesh R. Green synthesis and characterization of zinc oxide nanoparticles from Ocimum basilicum L. var. purpurascens Benth.-Lamiaceae leaf extract. Mat Lett 2014; 131: 16–18.

[15] Yuvakkumar R, Suresh J, Saravanakumar B, Nathanael AJ, Hong SI, Rajendran V. Rambutan peels promoted biomimetic synthesis of bioinspired zinc oxide nanochains for biomedical applications. Spectrochim. Acta A Mol Biomol Spect. 2015; 137: 250–258. 10.1016/j.saa.2014.08.022 25228035

[16] Samat NA, Nor RM. Sol–gel synthesis of zinc oxide nanoparticles using Citrus aurantifolia extracts. Ceramics Int. 2013; 39: 545–548.

[17] Thi TUD, Nguyen TT, Thi YD, Thi KHT, Phan BT, Pham KN. Green synthesis of ZnO nanoparticles using orange fruit peel extract for antibacterial activities. RSC Adv. 2020; 10: 23899–23907. 10.1039/d0ra04926c PMC9055061 35517333

[18] Kubmarawa D, Ajoku G, Enwerem N, Okorie D. Preliminary phytochemical and antimicrobial screening of 50 medicinal plants from Nigeria. Afr. J Biotech. 2007; 6: 1690–1696.

[19] Rao TN, Riyazuddin, Babji P, Ahmad N, Khan RA, Hassan I, et al. Green synthesis and structural classification of Acacia nilotica mediated-silver doped titanium oxide (Ag/TiO2) spherical nanoparticles: Assessment of its antimicrobial and anticancer activity. Saudi J Biol Sci. 2019; 26(7): 1385–1391. doi: 10.1016/j.sjbs.2019.09.005 31866742 PMC6904800

[20] Dalziel JM. Useful plants of west tropical Africa. Crown Agents for Oversea Government and Administration, London. 1956; pp101–110.

[21] Azwanida N. A review on the extraction methods use in medicinal plants, principle, strength and limitation. Med Aromat Plants. 2015; 4: 2167–0412.

[22] Elumalai K, Velmurugan S, Ravi S, Kathiravan V, Ashokkumar S. Green synthesis of zinc oxide nanoparticles using Moringa oleifera leaf extract and evaluation of its antimicrobial activity. 2015; 143:158–164. doi: 10.1016/j.saa.2015.02.011 25725211

[23] Mahamuni PP, Patil PM, Dhanavade MJ, Badiger MV, Shadija PG, Lokhande AC, et al. Synthesis and characterization of zinc oxide nanoparticles by using polyol chemistry for their antimicrobial and antibiofilm activity. Biochem Biophy Reports. 2019; 17: 71–80. doi: 10.1016/j.bbrep.2018.11.007 30582010 PMC6295600

[24] Barighat S, Ghannadnia M, Baghshahi S. Green synthesis of silver nanoparticles using the plant extract of Salvia spinosa grown *in vitro* and their antibacterial activity assessment. J Nanostructure Chem. 2019; 9: 1–9.

[25] Balouiri M, Sadiki M, Ibnsouda SK. Methods for *in vitro* evaluating antimicrobial activity: A review. J Pharm Ana. 2016; 6: 71–79. doi: 10.1016/j.jpha.2015.11.005 29403965 PMC5762448

[26] Loo YY, Chieng BW, Nishibuchi M, Radu S. Synthesis of silver nanoparticles by using tea leaf extract from Camellia sinensis. Int J nanomedicine. 2012; 7: 4263. doi: 10.2147/IJN.S33344 22904632 PMC3418103

[27] Kalishwaralal K, BarathManiKanth S, Pandian SRK, Deepak V, Gurunathan S. Silver nanoparticles impede the biofilm formation by Pseudomonas aeruginosa and Staphylococcus epidermidis. Colloids Sur B. 2010; 79: 340–344. doi: 10.1016/j.colsurfb.2010.04.014 20493674

[28] Kulshrestha S, Khan S, Hasan S, Khan M. E, Misba L, Khan AU. Calcium fluoride nanoparticles induced suppression of Streptococcus mutans biofilm: an *in vitro* and *in vivo* approach. App Microbiol Biotech. 2016; 100: 1901–1914. 10.1007/s00253-015-7154-4 26610805

[29] Muzammil S, Khurshid M, Nawaz I, Siddique MH, Zubair M, Nisar MA et al. Aluminium oxide nanoparticles inhibit EPS production, adhesion and biofilm formation by multidrug resistant Acinetobacter baumannii. Biofouling. 2020; 1–13. doi: 10.1080/08927014.2019.1708334 32529892

[30] Repetto G, Del Peso A, Zurita JL. Neutral red uptake assay for the estimation of cell viability/cytotoxicity. Nature protocols. 2008; 3: 1125. doi: 10.1038/nprot.2008.75 18600217

[31] Pallavicini P, Arciola C, Bertoglio F, Curtosi S, Dacarro G, D’Agostino A, et al. Silver nanoparticles synthesized and coated with pectin: An ideal compromise for anti-bacterial and anti-biofilm action combined with wound-healing properties. J colloid Interf Sci. 2017; 498: 271–281. doi: 10.1016/j.jcis.2017.03.062 28342310

[32] Selim YA, Azb MA, Ragab I, Abd El-Azim MH. Green Synthesis of Zinc Oxide Nanoparticles Using Aqueous Extract of Deverra tortuosa and their Cytotoxic Activities. Sci Rep. 2020; 10; 1–9. doi: 10.1038/s41598-019-56847-4 32103090 PMC7044426

[33] Yildiz FH, Schoolnik GK. Vibrio cholerae O1 El Tor: identification of a gene cluster required for the rugose colony type, exopolysaccharide production, chlorine resistance, and biofilm formation. Pro Nat Acad Sci. 1999; 96: 4028–4033. 10.1073/pnas.96.7.4028 PMC22414 10097157

[34] Baumann J. Prostaglandin synthetase inhibiting O_2-radical scavenging properties of some flavonoids and related phenolic compounds. Naunyn-Schmiedebergs Arch Pharmacol. 1979; 308: 27–32.

[35] Alekish M, Ismail ZB, Albiss B, Nawasrah S. *In vitro* antibacterial effects of zinc oxide nanoparticles on multiple drug-resistant strains of Staphylococcus aureus and Escherichia coli: An alternative approach for antibacterial therapy of mastitis in sheep Vet Wor. 2018; 11: 1428. 10.14202/vetworld.2018.1428-1432 PMC6247879 30532497

[36] Moghaddam AB, Moniri M, Azizi S, Rahim RA, Ariff AB, Saad WZ et al. Biosynthesis of ZnO nanoparticles by a new Pichia kudriavzevii yeast strain and evaluation of their antimicrobial and antioxidant activities. Molecules. 2017; 22: 872. doi: 10.3390/molecules22060872 28538674 PMC6152784

[37] Azam A, Ahmed AS, Oves M, Khan MS, Habib SS, Memic A. Antimicrobial activity of metal oxide nanoparticles against Gram-positive and Gram-negative bacteria: a comparative study. Int J nanomed. 2012; 7: 6003. doi: 10.2147/IJN.S35347 23233805 PMC3519005

[38] Jacob V, Rajiiv P. *In vitro* analysis: the antimicrobial and antioxidant activity of zinc oxide nanoparticles from Curcuma longa. Asian J Pharm Clin Res. 2019; 12: 200–204.

[39] Yang F, Wu W, Chen S, Gan W. The ionic strength dependent zeta potential at the surface of hexadecane droplets in water and the corresponding interfacial adsorption of surfactants, Soft Matter. 2017; 13: 638–646. doi: 10.1039/c6sm02174c 27991633

[40] Liang S, Jiao X., Tan X., Zhu J., Effect of solvent film and zeta potential on interfacial interactions during optical glass polishing, Appl. Opt. 2018; 57: 5657–5665. doi: 10.1364/AO.57.005657 30118078

[41] Imran M, Iqbal MM, Iqbal J, Shah NS, Khan ZUH, Murtaza B, et al (2021): Synthesis, characterization and application of novel mno and cuo impregnated biochar composites to sequester arsenic (As) from water: modeling, thermodynamics and reusability. Journal of Hazardous Materials 401, 123338. doi: 10.1016/j.jhazmat.2020.123338 32634661

[42] Agarwal H, Nakara A, Menon S, Shanmugam V. Eco-friendly synthesis of zinc oxide nanoparticles using Cinnamomum Tamala leaf extract and its promising effect towards the antibacterial activity. J Drug Deliv Sci and Technol; 2019; 53: 101212

[43] Nithya KS and Kalyanasundharam. Effect of chemically synthesis compared to biosynthesized ZnO nanoparticles using aqueous extract of C. halicacabum and their antibacterial activity, OpenNano. 2019; 4: 100024.

[44] Zare E, Pourseyedi S, Khatami M, Darezereshki E. Simple biosynthesis of zinc oxide nanoparticles using nature’s source, and it’s *in vitro* bio-activity. J Mol Str. 2017; 1146: 96–103.

[45] Naseer M, Aslam U, Khalid B, Chen B. Green route to synthesize Zinc Oxide Nanoparticles using leaf extracts of Cassia fistula and Melia azadarach and their antibacterial potential. Sci Rep. 2020; 10: 1–10. doi: 10.1038/s41598-019-56847-4 32493935 PMC7270115

[46] Cox SD, Markham J. Susceptibility and intrinsic tolerance of Pseudomonas aeruginosa to selected plant volatile compounds. J App Microbiol. 2007; 103: 930–936. doi: 10.1111/j.1365-2672.2007.03353.x 17897196

[47] Arakha M, Saleem M, Mallick BC, Jha S. The effects of interfacial potential on antimicrobial propensity of ZnO nanoparticle. Sci. Rep. 2015; 5: 9578. doi: 10.1038/srep09578 25873247 PMC4397836

[48] Peng X, Palma S, Fisher, NS, Wong SS. Effect of morphology of ZnO nanostructures on their toxicity to marine algae. Aquat. Toxicol. 2011; 102: 186–196. doi: 10.1016/j.aquatox.2011.01.014 21356181

[49] Lara HH, Ayala-Núñez NV, Turrent LdCI, Padilla CR. Bactericidal effect of silver nanoparticles against multidrug-resistant bacteria. Wor J Microbiol Biotech. 2010; 26: 615–621.

[50] Atkinson A, Winge DR. Metal acquisition and availability in the mitochondria. Chem. Rev. 2009; 109: 4708–4721. doi: 10.1021/cr900006y 19522505 PMC3881427

[51] Vanaja M, Gnanajobitha G, Paulkumar K, Rajeshkumar S, Malarkodi C, Annadurai G. Phytosynthesis of silver nanoparticles by Cissus quadrangularis: influence of physicochemical factors. J Nanostructure Chem. 2013; 3: 17.

[52] Hajipour MJ, Fromm KM, Ashkarran AA, de Aberasturi DJ, Larramendi IR, Rojo T et al. Antibacterial properties of nanoparticles. Tren Biotech. 2012; 30: 499–511. doi: 10.1016/j.tibtech.2012.06.004 22884769

[53] Jones BV, Young R, Mahenthiralingam E, Stickler DJ. Ultrastructure of Proteus mirabilis swarmer cell rafts and role of swarming in catheter-associated urinary tract infection. Inf Immun. 2004; 72: 3941–3950. doi: 10.1128/IAI.72.7.3941-3950.2004 15213138 PMC427392

[54] Kostakioti M, Hadjifrangiskou M, Hultgren SJ. Bacterial biofilms: development, dispersal, and therapeutic strategies in the dawn of the postantibiotic era. Cold Spring Harbor perspectives in medicine. 2013; 3: a010306. doi: 10.1101/cshperspect.a010306 23545571 PMC3683961

[55] Siddique MH, Aslam B, Imran M, Ashraf A, Nadeem H, Hayat S. Effect of Silver Nanoparticles on Biofilm Formation and EPS Production of Multidrug-Resistant Klebsiella pneumoniae. BioMed Res Int. 2020. doi: 10.1155/2020/6398165 32382563 PMC7189323

[56] Al-Shabib A, Husain FM, Hassan I, Khan MS, Ahmed F, Qais FA et al. Biofabrication of zinc oxide nanoparticle from Ochradenus baccatus leaves: broad-spectrum antibiofilm activity, protein binding studies, and *in vivo* toxicity and stress studies. J Nanomaterials. 2018; 20181–14.

[57] Lorian V. Effect of low antibiotic concentrations of bacteria: effects on ultrastructure, virulence, and susceptibility to immunodefenses. Antibio Lab Med. 1991; 493–555.

[58] Xia L, Huang R, Li Y, Song S. The effect of growth phase on the surface properties of three oleaginous microalgae (Botryococcus sp. FACGB-762, Chlorella sp. XJ-445 and Desmodesmus bijugatus XJ-231). Plos one. 2017; 12: e0186434. doi: 10.1371/journal.pone.0186434 29045481 PMC5646804

[59] Bharathi D, Bhuvaneshwari V. Synthesis of zinc oxide nanoparticles (ZnO NPs) using pure bioflavonoid rutin and their biomedical applications: antibacterial, antioxidant and cytotoxic activities. Res Chem Int. 2019; 45: 2065–2078.

[60] Kanagaraj P, Arunachalam R, Devaraj B, Subbiah R. Anti-oxidant activity, phytochemical screening and HPLC profile of rare endemic Cordia diffusa. J King Saud Univer-Sci. 2019; 31(4): 724–727.

[61] Bisht G, Rayamajhi S. ZnO nanoparticles: a promising anticancer agent. Nanobiomedicine. 2016; 3: 3–9. doi: 10.5772/62337 29942384 PMC5998263

[62] Seaman SJ, Dyar MD, Marinkovic N, Dunbar NW. An FTIR study of hydrogen in anorthoclase and associated melt inclusions. Am Mineralo. 2006; 91: 12–20.

